# Individual Differences in the Association of Lifestyle with Cardiometabolic Risk in Middle-Aged Koreans Based on Traditional Korean Medicine

**DOI:** 10.3390/ijerph192214963

**Published:** 2022-11-14

**Authors:** Younghwa Baek, Kihyun Park, Kyoungsik Jung, Siwoo Lee

**Affiliations:** KM Data Division, Korea Institute of Oriental Medicine, Daejeon 34054, Korea

**Keywords:** cardiometabolic risk, lifestyle, physical activity, sleep, eating index, Sasang constitution

## Abstract

Cardiometabolic (CM) risk differs in morbidity and disease progression depending on lifestyle and individual characteristics. This cross-sectional study aimed to analyze the association between CM risk and lifestyle and examine whether this association varies based on Sasang constitution (SC), a Korean medicine classification. Data were analyzed from 1996 individuals participating in the Korean Medicine Daejeon Citizen Cohort study. Individuals with CM risk had two to five CM risk factors. Lifestyle factors included physical activity, sleep duration, and eating index. SC types were Taeeum-in (TE) and non-TE. We estimated the odds ratio (OR) and 95% confidence interval for CM risk based on SC and lifestyle factors. Among the participants, 33.9% had a CM risk, and the TE and non-TE groups were 26.2% and 7.7%, respectively. In the TE group, CM risk was associated with low physical activity (OR, 1.63) and moderate eating index (OR, 1.41). In the non-TE group, CM risk was associated with ≥8 h of sleep (OR, 1.87) and marginally associated with ≤6 h of sleep. In the TE group alone, CM risk was significantly associated with lifestyle patterns that combined the three lifestyle factors and was approximately two-fold higher in patterns involving less physical activity. The effects of lifestyle on CM risk differed across SC types. To decrease the burden of cardiovascular diseases in middle-aged adults, we recommend an individualized management strategy of healthy lifestyle interventions for cardiovascular risks.

## 1. Introduction

Cardiometabolic (CM) risk encompasses a cluster of metabolic and cardiovascular abnormalities, such as abdominal obesity, insulin resistance, hypertension, dyslipidemia, and atherosclerosis [1]. CM risk is closely linked to cardiovascular disease (CVD) and type 2 diabetes mellitus (DM) [2]. It poses a significant burden worldwide and exacerbates the public health crisis of coronavirus disease (COVID-19) [3]. Between 1980 and 2014, the prevalence of CM risk factors rose over time throughout the United States; in particular, the prevalence of obesity and DM increased two- to four-fold [4]. Moreover, owing to population aging, approximately 25% of adults under 65 years receiving health management have multimorbidity, which includes one or more chronic conditions or CM risk factors [5]. Hence, employing comprehensive prevention management is important for individuals with CM risk factors who have yet to develop a chronic condition or who only have mild conditions.

Major lifestyle factors, such as physical activity, sleep, and diet, are modifiable factors that substantially contribute to individuals’ CM risk, cardiovascular health, and subsequent mortality [6,7,8]. Over the past two decades, young adults aged 18–45 years have been exposed to more health risk factors, such as obesity, poor diet, and physical inactivity; in addition, as the current young adult population ages, new waves of CVD in the population will be of concern [9]. However, adopting a healthy lifestyle offers more health benefits in young adults and lowers the risk of pre-diagnosed CVD and multimorbidity. A meta-analysis of 20 cohort studies showed that combined healthy lifestyle habits were more beneficial for CVD prevention in adults < 50 years than older adults [10]. Investigators in a cohort study of seven European countries similarly reported that a healthy lifestyle in adults in their 40s and 50s lowered the risk of multimorbidity [11] and that the importance of a particular factor in disease progressions, such as the development of a single disease, multimorbidity, and mortality in disease-free middle-aged adults varies depending on the stage of the disease. Clinical parameters, such as blood pressure, determine the risk of the initial onset of CVD; however, lifestyle factors are potentially more predictive of multimorbidity and progression to mortality in middle-aged adults with CVD [12].

The effects of a healthy lifestyle on health outcomes may differ based on individual characteristics. Sasang constitutional medicine (SCM) in Korean medicine classifies individuals based on their physical, psychological, and physiological characteristics into four different Sasang constitution (SC) types: Taeyang-in, Taeeum-in (TE), Soeum-in (SE), and Soyang-in (SY) [13]. The four SC types have been classified according to skewed balance between two visceral systems in specific formal pairs, namely the lung-liver pair (consumption and strong storage of Qi and body fluid) and the spleen-kidney pair (intake and waste excretion of raw material) by the viewpoint of SCM. The TY type has a hyperactive lung and a hypoactive liver system, whereas the TE type has a hyperactive liver and a hypoactive lung system. The SY type has a hyperactive spleen and a hypoactive kidney system, whereas the SE type has a hyperactive kidney and a hypoactive spleen system [13]. Due to the different characteristics of each type, disease vulnerability and treatment differ across SC types, and the four SC types also differ in modifiable factors for preventive health management or potential risk factors (e.g., lifestyle) [14]. Recent studies have shed light on the association between lifestyle and chronic disease in SC. One study confirmed that the effects of various lifestyle factors on hypertension vary across SC types. Being overweight and excessive alcohol consumption were associated with hypertension risk in the SY type, and in addition to the preceding factors, physical inactivity in the TE type [15]. Other studies that examined the association between individual lifestyle and chronic conditions report an association between sleep quality and obesity in the SY and TE types [16] and that an unhealthy diet, such as poor diet, poor nutrition, and low vegetable intake, is associated with the prevalence of CM diseases [17,18]. However, few studies have multilaterally examined the relationship between various lifestyle factors and CM risk, based on SC type, in individuals in their 30s to 50s who would reap substantial benefits from adopting a healthy lifestyle. With this context, this study aimed to investigate the association between lifestyle—primarily focusing on physical activity, sleep, and eating—and CM risk status, defined as having two or more CM risk factors, among the middle-aged adult population in Korea and examine whether the effects of these lifestyle factors would differ, based on SC type.

## 2. Materials and Methods

### 2.1. Study Design and Participants

This study was a cross-sectional analysis of baseline data from the Korean Medicine Daejeon Citizen Cohort (KDCC) study conducted from 2017 to 2019. The design of the KDCC has previously been described in detail [19]. In brief, the KDCC is a prospective ongoing cohort study of the community-based population in Korea to assess the associations between genetic factors, lifestyle factors, and chronic diseases based on the concept of traditional Korean medicine. The participants included individuals aged ≥ 30 years and ≤55 years who were residents of Daejeon, Korea, but excluded those with a diagnosis of cancers or any CVD, such as myocardial infarction, angina, or stroke. Two thousand participants were enrolled in the KDCC study at baseline. In the present study, 1996 participants were included in the analysis, excluding participants with missing values for major variables (no lifestyle variables = 3, no clinical outcomes = 1).

### 2.2. Variables

#### 2.2.1. Lifestyle Factors

Lifestyle factors included physical activity, sleep duration, and eating index; data were obtained from structured questionnaires using face-to-face interviews.

Physical activity was assessed using the Korean Global Physical Activity Questionnaire (GPAQ), developed by the World Health Organization (WHO) [20,21]. The GPAQ assesses the amount of physical activity by domain, such as activity at work, travel to and from places, and recreational activities. It also measures the intensity levels of physical activity by converting it to a metabolic equivalent task (MET), which is commonly used in the analysis of physical activity. Based on the GPAQ analysis guidelines, GPAQ scores are divided into three levels of physical activity considering total physical activity MET-min/week, calculated using 4.0 METs for moderate activity and 8.0 METs for vigorous activity, and the days and minutes of physical activity. In this study, physical activity was classified as “high”, “moderate”, or “low”.

Sleep duration was assessed using the question, “How many hours did you sleep every day on average in the past month?” The responses were divided into 7 h of sleep (“good sleep”; the reference), ≤6 h, and ≥8 h of sleep.

The eating index (EI) was surveyed using the semi-quantitative Food Frequency Questionnaire, a modified version based on items from the 2011 Korea Health and Nutrition Examination Survey [22]. The reported frequency (nine categories ranging from “never” to “three times a day”) and portion size (three or four specified portion sizes) of each food item were converted to daily gram intake of macronutrients and total energy using the Computer-Aided Nutritional Analysis Program (CAN Pro, Version 5.0, The Korean Nutrition Society, 2015, Seoul, Korea). With reference to the method of calculating the Korean Healthy Eating Index [23], the EI was calculated with a range of 0–100, in which a higher score indicated better diet quality. The EI values were classified based on quartile: high EI (≥58.5; ≥75%), moderate EI (44–58.5; 25–74%), and low EI (0–43; <25%).

#### 2.2.2. Sociodemographic Variables

Sociodemographic characteristics of the study population, such as sex, age, education level, marital status, smoking, alcohol consumption, and disease history, were also assessed via face-to-face interviews. Education level was classified as less than high school and college and higher level based on the following categories: less than elementary school, middle school, high school, and above college. Marital status was classified as “married” or “unmarried”, which included single, separated, divorced, widowed, etc. With regard to the smoking status, participants who had not smoked 100 or more cigarettes in their lifetimes were considered “never smokers”; participants who had smoked 100 or more cigarettes in their lifetimes were considered “current smokers” or “former smokers” with the question “Do you smoke now?”. Alcohol consumption was divided into three categories (“nondrinker”, “responsible drinker”, and “hazardous drinking”). The average volume of alcohol per day (g/day) was calculated based on drinking frequency (times/day), the volume of alcohol per seating (drinks/seating), and alcohol content (g/drink) for each type of alcohol (i.e., rice wine, refined rice wine, wine, soju, beer, spirits, and others). Regarding the average volume of alcohol per day [24], “nondrinker” refers to a person who has not had an alcohol-containing drink, whereas “responsible drinker” refers to women who drink 0.1–19.99 g/day and men who drink 0.1–39.99 g/day. Hazardous drinking refers to alcohol consumption ≥ 20 g/day in women and 40 g/day in men. Disease history (e.g., hypertension, DM, dyslipidemia) was surveyed through a self-report by asking participants whether they had been diagnosed with related-diseases by a physician.

#### 2.2.3. Anthropometric and Biochemical Variables

Anthropometric measurements were taken to the nearest 0.1 kg or 0.1 cm. Height and weight were measured, with participants dressed lightly without shoes. BMI was calculated as weight in kilograms divided by height in meters squared (k/m^2^). Waist circumference (WC) was measured at the level of the umbilicus at the end of a normal breath using a tape measure (Rollfix; Hoechstmass-Balzer, Sulzbach, Germany). Blood pressure (BP) was measured once using an automatic blood pressure cuff (FT-500R PLUS; Jawon Medical Company, Seoul, Korea) and was measured again after 5–10 min of rest; the mean value of the two measurements was used for analysis. Triglyceride (TG), high-density lipoprotein cholesterol (HDL-C), and fasting plasma glucose (FPG) levels were recorded with blood tests obtained after overnight fasting and were measured using an enzymatic colorimetric method (ADVIA 1800; Siemens, New York, NY, USA) at the Seoul Clinical Laboratories (Seoul, Korea).

### 2.3. Definition of Cardiometabolic Risk Factors

Cardiometabolic risk factors were previously categorized using the National Cholesterol Education Program-Adult Treatment Panel III (NCEP-ATP III) guidelines [25]. The CM status in this study was divided into the “CM risk group” (i.e., participants had two or more of the following clinical criteria) and the “normal group” (i.e., participants had zero to one of the cardiometabolic risk factors): (1) increased WC with cut-off points specific to South Koreans (WC ≥ 90 cm in men and ≥85 cm in women); (2) elevated BP, systolic BP (SBP) ≥ 130 or diastolic BP (DBP) ≥ 85 mmHg or antihypertensive medication use in individuals with a history of hypertension; (3) elevated TG levels ≥ 150 mg/dL or specific treatment for this lipid abnormality; (4) reduced HDL-C level, 40 mg/dL in men and 50 mg/dL in women or drug treatment for this lipid abnormality; and (5) elevated FPG level ≥ 100 mg/dL or antidiabetic medication use in individuals with a history of DM.

### 2.4. Sasang Constitution Type

The 15-item Korean Sasang Constitutional Diagnostic Questionnaire (KS-15) [26] was used to assess the individual constitution of the participants. The KS-15 comprises one item on anthropometric awareness of height and weight, six questions on personality (broad-minded/delicate, quick/slow acting, active/passive, extraverted/introverted, masculine/feminine, excitable/rational), and eight symptom-related questions on physiological functions (adequate digestion, appetite, much sweat, feeling after sweating, abdominal tension during a bowel movement, urination at night during sleep time, cold and heat dislike, temperature preference when drinking water). The KS-15 is a short-form SC classification tool with established clinical relevance and high reliability (Cronbach’s alpha = 0.630, test–retest reliability = 0.469–0.734) and classifies individuals into TE, SE, or SY types [26,27]. We defined the two constitution types as the “TE group” or “non-TE (SE and SY) group” to compare the differences in lifestyle factors associated with cardiometabolic risk factors.

### 2.5. Statistical Analysis

We analyzed the association between lifestyle and CM risk by stratifying individuals into TE and non-TE types based on SCM, which considers individuals’ physiological and physical characteristics. Descriptive analyses of the participants are presented in four groups based on their SC type and CM risk status, TE/non-TE with CM risk group or TE/non-TE with the normal group. Four group differences were evaluated using chi-square tests for categorical variables and a one-way analysis of variance for continuous variables. Differences in lifestyle factors between the groups were compared using the mean values and categories of physical activity, sleep duration, and EI. The associations of individual lifestyle factors with CM risk status and five CM risk factors were examined using logistic regression models based on the SC type. In addition, the association between lifestyle patterns and CM risk status was examined using logistic regression models. Lifestyle patterns were divided into “unhealthy” and “healthy” lifestyles based on the following features: low physical activity (i.e., low GPAQ score) versus healthy physical activity (i.e., moderate and high GPAQ scores), poor sleep duration (i.e., ≤6 h or ≥8 h) versus healthy sleep duration (i.e., 7 h), and poor EI (i.e., low and moderate EI) versus healthy EI (i.e., high EI). These lifestyle categories were combined to generate eight lifestyle patterns. The association between the remaining lifestyle patterns and CM risk was analyzed with reference to the healthy lifestyle pattern combined into the healthy lifestyle in all three factors. The odds ratio (OR) and 95% confidence interval (CI) are presented using multivariate logistic regression, adjusted for sex, age, BMI, education level, marital status, smoking status, alcohol consumption, and disease history. We conducted all analyses using SAS 9.4 (SAS Institute, Cary, NC, USA). A *p*-value of <0.05 was significant.

## 3. Results

### 3.1. The Participants’ General Characteristics

Of the 1996 participants, the CM risk group comprised 33.9%; among all participants, the TE group with CM risk accounted for 26.2%, whereas the non-TE group with CM risk accounted for 7.7%. Except for education level, all variables significantly differed among the four groups (Table 1).

### 3.2. Association between Each Lifestyle and CM Risk by SC Type

CM risk was associated with physical activity and EI in the TE group and with sleep duration in the non-TE group (Table 2). In the TE group, the odds for CM risk were 1.63 times higher for individuals with low physical activity than for individuals with high physical activity (OR, 1.63; 95% CI, 1.13–2.38 in Model 2). In the sex- and age-adjusted Model 1, the prevalence of CM risk in the TE group was 1.41 times higher for individuals with a moderate EI than for individuals with a high EI (OR, 1.41; 95% CI, 1.02–1.95), but this significance was lost in Model 2. In the non-TE group, the odds for CM risk were 1.87 times higher for individuals with ≥8 h of sleep than for individuals with 7 h of sleep (OR, 1.87; 95% CI, 1.07–3.29 in Model 2). A marginal association existed between ≤6 h of sleep and CM risk (OR, 1.53; 95% CI, 0.98–2.39 in Model 2).

### 3.3. Association between Lifestyle Patterns and CM Risk

**Figure 1:**
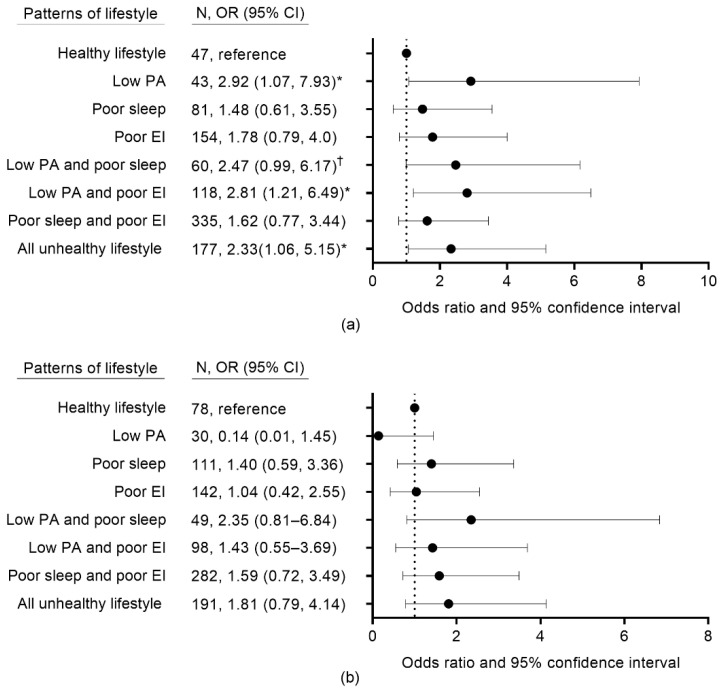
Associations between lifestyle patterns and CM risk based on SC type: (**a**) TE group; (**b**) Non-TE group. CM, cardiometabolic; SC, Sasang constitution; OR, odds ratio; CI, confidence interval; TE, Taeeum-in; PA, physical activity; EI, eating index. OR is adjusted for sex, age, body mass index, education, marriage, smoking, alcohol consumption, and disease history. Individual lifestyle factors are classified as unhealthy versus healthy. The factors were combined to generate patterns: PA is classified as low PA versus healthy PA (i.e., moderate and high PA), sleep duration is classified as poor sleep duration (≤6 h and ≥8 h) versus healthy sleep duration (7 h); and EI is classified as poor EI (i.e., low and moderate EI) versus healthy EI (i.e., high EI). Healthy lifestyle includes high PA, healthy sleep, and healthy EI. * *p* < 0.05, ^†^ *p* < 0.1.

With regard to eight lifestyle patterns comprising various combinations of healthy and unhealthy lifestyles for each lifestyle factor, the prevalence of poor sleep and low EI pattern was highest in the TE group and the non-TE group. With reference to the pattern of a healthy lifestyle, the odds for CM risk were higher in the pattern of low physical activity (OR, 2.92; 95% CI, 1.07–7.93), the pattern of low physical activity and poor EI (OR, 2.81; 95% CI, 1.21–6.49), and patterns of low physical activity, poor sleep, and poor EI (OR, 2.33; 95% CI, 1.06–5.15) in the TE group. The pattern of low physical activity and poor sleep was marginally associated with CM risk in this group (OR, 2.47; 95% CI, 0.99–6.17). In the non-TE group, no significant associations existed between lifestyle patterns and CM risk (Figure 1).

### 3.4. Associations between Each Lifestyle and Individual CM Risk Factors

Table 3 shows the associations between each lifestyle and the five CM risk factors based on SC type. In the TE group, a high WC was associated with low physical activity (OR, 1.7; 95% CI, 1.13–2.56), and high glucose was associated with low physical activity (OR, 1.93; 95% CI, 1.06–3.51) and moderate physical activity (OR, 2.04; 95% CI, 1.14–3.63). In the non-TE group, physical activity, sleep, and EI were all associated with individual CM risk factors. A low HDL-C level was associated with moderate physical activity (OR, 1.76; 95% CI, 1.09–2.83) and long sleep duration (OR, 1.84; 95% CI, 1.09–3.1). Short sleep was associated with a high WC (OR, 2.14; 95% CI, 1.01–4.5), and moderate EI was associated with a high TG level (OR, 1.85; 95% CI, 1.18–2.89).

## 4. Discussion

This study explored the associations between lifestyle factors (physical activity, sleep, and eating) and CM risk based on SC type in middle-aged adults in Korea. One key finding of this study was that the lifestyle factors associated with CM risk differed across SC types. CM risk was associated with low physical activity and moderate EI among the TE types and undesirable sleep duration among the non-TE types. In addition, we confirmed that the association of CM risk with the combination of physical activity, sleep duration, and EI was primarily evident in the TE type. The important result was that, among TE individuals, the prevalence of CM risk was approximately two-fold higher for lifestyle patterns of low physical activity with or without poor sleep and/or poor EI. Finally, we observed that physical activity, sleep duration, and EI had different effects on each of the five CM risk factors across the SC types. The results of this study indicate that a strategy focusing on adherence to priority healthy lifestyles should be used to improve CM risk status in consideration of individuals’ characteristics as well as the physiological and pathological statuses of CM risk factors.

In this study, CM risk was defined as having two or more components of metabolic syndrome, as delineated in the NECP-ATP III guidelines [25]. From a clinical perspective, metabolic syndrome is associated with cardiovascular outcomes, which is supported by a high level of evidence [28]. Furthermore, developing desirable lifestyle practices before the onset of CVD has more positive effects on young adults [10,11,12]. We included the concept of the pre-disease stage, a high-risk status proceeding CVD, and metabolic syndrome [29]. In this study, the prevalence of CM risk was approximately one in three people, was two-fold greater in men (51%) than in women (26%), and increased with age. The prevalence of metabolic syndrome in Korea was approximately 20.3% in 2015, which was similar throughout the years since 2007 [30]. However, the prevalence in men aged 19–49 years has steadily increased over the years, which highlights the need for the management of metabolic syndrome in young and middle-aged men [30]. In addition, approximately 20–25% of adults had two or more metabolic syndrome components [31,32], and the rate progressively increased across the age groups [32]. These results were similar to the rate observed in our study.

In the TE group, low physical activity was associated with CM risk with reference to high physical activity and no CM risk. In one study [15], TE types who engaged in physical activity had a 25% lower prevalence of hypertension than those with physical inactivity. Furthermore, TE types generate relatively less heat and thus have a slower metabolism and weaker lung functions, which results in weak maximal aerobic capability and consequently facilitates weight gain [33,34]. Therefore, TE-type individuals should engage in sufficient aerobic exercise, and practicing optimal physical activity levels is essential for their CM risk management. Moreover, in light of our results demonstrating that low physical activity in TE individuals increases the odds for a higher WC and glucose levels two-fold, focusing on the physical activity status in TE patients with a clinical risk for abdominal obesity and DM is important.

An unhealthy diet is strongly associated with a substantial increase in the incidence of metabolic syndrome and CVD [7]. Our study confirmed that moderate EI was associated with CM risk with reference to high EI and no CM risk in the TE group after adjusting for sex and age. A previous study reported that poor nutrition increases the prevalence of metabolic syndrome by approximately two-fold [17] and that high vegetable consumption lowers the prevalence of CVD by up to 63–86% in TE individuals [18]. Considering that TE individuals possess a fat mass and obesity-associated (FTO) gene [35] and are physiologically more vulnerable to weight gain because of an imbalance of energy conservation [34], a healthy diet is an important lifestyle factor, along with adequate physical activity, to prevent CM risk.

We observed that inappropriately short or long sleep durations are associated with CM risk, that short sleep duration is associated with abdominal obesity, and that long sleep duration is associated with reduced HDL-C levels in non-TE individuals. Less than six hours of sleep has been associated with metabolic syndrome and increased WC, and a sleep duration of 10 h or longer has been linked to reduced HDL-C levels in women [36]; this is similar to our results. In SCM, sleep is an important clinical indicator for the current health status and the assessment of interventions and prognosis; sleep states also vary across SC types [37]. Investigators of an experimental study [38], which limited sleep duration, reported that SY individuals are more sensitive to sleep restriction than TE individuals and have a slower recovery of body composition altered by sleep deprivation. Furthermore, poor sleep quality has been associated with obesity in SY and SE individuals [16]. This finding suggests that the relationship between sleep and health outcomes differs based on individual characteristics, and that sleep is an important lifestyle factor in non-TE individuals. However, we did not distinguish between SE and SY individuals because of the low prevalence of CM risk among the non-TE types, and short sleep duration was marginally significantly associated with CM risk. This issue should be examined in future studies.

In addition, in non-TE individuals, an association existed between moderate physical activity and low HDL-C and between moderate EI and increased TG. Clinical practice guidelines for different SC types recommend light exercise, such as walking and stretching, as opposed to vigorous exercise, for SE individuals and more active exercise, such as hiking and jogging, for SY individuals. Moreover, helpful and nonhelpful foods differed between the SC types [14]. A few studies have described a relationship between each lifestyle factor and CM risk in non-TE individuals, but the findings were not concrete [15,17]. However, our study presents clinical evidence for assessing physical activity, sleep duration, and EI in non-TE individuals at risk of dyslipidemia and abdominal obesity. Subsequent studies should also analyze the relationship between lifestyle and CM risk in more depth in consideration of the specific SC types among non-TE individuals.

Adhering to several healthy lifestyle practices concurrently results in more positive effects on outcomes relevant to metabolic syndrome and CVD [10,11]. A healthy lifestyle was linearly correlated with a life expectancy free of chronic diseases, and an optimal healthy lifestyle added approximately 9 years to healthy life years [39]. Furthermore, a meta-analysis of the effects of lifestyle modification reported that lifestyle interventions were approximately twice as effective in resolving metabolic syndrome and reducing the severity of fasting glucose, WC, BP, and TG in individuals with metabolic syndrome [40]. Our study showed an association between eight lifestyle patterns, primarily involving compliance with multiple lifestyle recommendations and CM risk in TE individuals. Moreover, the prevalence of CM risk increased by 2.33–2.92 times for low physical activity alone or physical activity in combination with poor sleep and/or poor EI in TE individuals. Hence, physical activity is a priority lifestyle factor contributing to the prevention of CM risk in middle-aged individuals with TE.

This study had several limitations. First, we conducted a cross-sectional analysis of only the baseline data from a prospective cohort study. Therefore, we could not evaluate the causality between lifestyle factors and CM risk. Second, although we used a widely used, validated structured questionnaire to assess lifestyle factors, the questionnaire is self-reported; thus, the collected data are vulnerable to bias. Finally, the study was conducted on young adults. The data of patients with CVD are relatively scarce in this population, and the estimates of some results had a wide CI [9]. Therefore, caution is necessary when interpreting the results.

Nevertheless, our study had several strengths. To the best of our knowledge, this study is the first to analyze the relationships between lifestyle and CM risk and specific CM risk factors in the Korean middle-aged population based on SC type. We presented evidence supporting that a healthy lifestyle is a classic modifiable factor with a pivotal role in preventing CM risk in middle-aged adults. Furthermore, we proposed a comprehensive view for devising individualized health management plans focused on cultivating a healthy lifestyle in daily living.

## 5. Conclusions

This study examined the associations between lifestyle factors—namely, physical activity, sleep, diet, and individual CM risk factors—and the differences in these associations based on SC type in the Korean middle-aged population. Appropriate physical activity and a healthy diet are crucial for the TE type to prevent and manage the CM risk. Appropriate sleep duration is an important lifestyle practice for non-TE individuals. In addition, the associations between lifestyle factors and CM risk factors should be assessed depending on the stage of cardiovascular and metabolic disorders. For example, a recommendation is that TE types engage in good levels of physical activity when they are at risk of abdominal obesity and DM, whereas non-TE types should be assessed for physical activity, sleep duration, and EI when they are at risk of dyslipidemia or abdominal risk. Our results provide insights into healthy lifestyle practices for CVD prevention by identifying the key lifestyle factors associated with CM risk based on an individual’s characteristics.

## Figures and Tables

**Table 1:** Demographic and clinical characteristics of the participants.

Variable		CM Risk Group (n = 676)		Normal Group (n = 1320)		*p*-Value
		TE	Non-TE	TE	Non-TE	
		(n = 522)	(n = 154)	(n = 493)	(n = 827)	
**Age (y)**	30–44	235 (45)	42 (27.3)	290 (58.8)	429 (51.9)	<0.001
	45–55	287 (55)	112 (72.7)	203 (41.2)	398 (48.1)	
**Sex**	Men	243 (46.6)	70 (45.5)	131 (26.6)	166 (20.1)	<0.001
	Women	279 (53.5)	84 (54.6)	362 (73.4)	661 (79.9)	
**Married ** **status ^1^**	Unmarried	60 (11.6)	12 (7.8)	89 (18.1)	114 (13.8)	0.003
	Married	458 (88.4)	141 (92.2)	404 (82)	711 (86.2)	
**Education ^1^**	Under high school	201 (38.9)	57 (37.3)	160 (32.6)	293 (35.6)	0.213
	College and higher	316 (61.1)	96 (62.8)	331 (67.4)	531 (64.4)	
**Smoking ** **status**	Never	356 (68.2)	107 (69.5)	409 (83)	716 (86.6)	<0.001
	Former	71 (13.6)	17 (11)	38 (7.7)	37 (4.5)	
	Current	95 (18.2)	30 (19.5)	46 (9.3)	74 (9)	
**Alcohol ** **consumption**	Nondrinker	184 (35.3)	58 (37.7)	191 (38.7)	357 (43.2)	0.001
	Responsible	260 (49.8)	71 (46.1)	258 (52.3)	395 (47.8)	
	Hazardous	78 (14.9)	25 (16.2)	44 (8.9)	75 (9.1)	
**Disease ** **history (yes)**	Hypertension	84 (16.1)	21 (13.6)	12 (2.4)	22 (2.7)	<0.001
	Diabetes	28 (5.4)	6 (3.9)	4 (0.8)	7 (0.9)	<0.001
	Dyslipidemia	81 (15.5)	16 (10.4)	25 (5.1)	49 (5.9)	<0.001
**BMI (kg/m^2^)**		28.2 ± 3.1 ^a^	23.3 ± 1.7 ^c^	25.3 ± 2.3 ^b^	21.5 ± 2 ^d^	<0.001
**CM risk factor**	WC (cm)	92.5 ± 7.9 ^a^	82.0 ± 6.3 ^c^	83.9 ± 6.4 ^b^	76 ± 6.3 ^d^	<0.001
	SBP (mmHg)	126.5 ± 15.6 ^a^	126.4 ± 17 ^a^	113.8 ± 12.2 ^b^	111 ± 12.5 ^c^	<0.001
	DBP (mmHg)	81.1 ± 12.7 ^a^	80 ± 11.8 ^a^	71.1 ± 9.9 ^b^	68.9 ± 9.9 ^c^	<0.001
	TG (mg/dL)	198.3 ± 158.8 ^a^	227.1 ± 238.9 ^a^	97.8 ± 43.9 ^b^	93.3 ± 53.8 ^c^	<0.001
	HDL-C (mg/dL)	47.6 ± 10.3 ^a^	48.3 ± 12.2 ^a^	58.9 ± 11.8 ^b^	63.1 ± 13.4 ^c^	<0.001
	FPG (mg/dL)	90.7 ± 22.7 ^a^	92.7 ± 28 ^a^	81.4 ± 7.3 ^b^	80.1 ± 8.4 ^b^	<0.001

BMI, body mass index; CM, cardiometabolic; WC, waist circumference; SBP, systolic blood pressure; DBP, diastolic blood pressure; TG, triglyceride; HDL-C, cholesterol; FPG, fasting plasma glucose; TE, Taeeum-in. Data are presented as the n (%) or the mean ± standard deviation. The *p* values were obtained from chi-square tests for categorical variables and one-way analysis of variance for continuous variables among the four groups. ^1^ The value includes missing values. ^a–d^ Different letters indicate statistically significant differences (*p* < 0.05), analyzed by using analysis of variance, followed by Bonferroni’s multiple comparison post hoc test.

**Table 2:** Associations between each lifestyle and CM risk by SC types.

	TE Group			Non-TE Group		
	No. CM Risk (No/Yes)	Model 1	Model 2	No. CM Risk (No/Yes)	Model 1	Model 2
Physical activity						
High	150/150	reference		249/45	reference	
Moderate	165/152	0.98 (0.71–1.36)	1.02 (0.69–1.51)	268/51	1.36 (0.85–2.17)	1.31 (0.80–2.15)
Low	178/220	1.27 (0.93–1.74)	**1.63 (1.13–2.38)** *	310/58	1.42 (0.9–2.24)	1.34 (0.83–2.16)
Sleep duration						
7 h	174/188	reference		302/46	reference	
≤6 h	224/258	1.06 (0.8–1.41)	0.92 (0.66–1.29)	380/76	1.4 (0.91–2.14)	1.53 (0.98–2.39) **^†^**
≥8 h	95/76	0.81 (0.56–1.19)	0.88 (0.56–1.39)	145/32	**1.84 (1.08–3.14)** *	**1.87 (1.07–3.29)** *
Eating index						
High	125/106	reference		227/41	reference	
Moderate	242/277	**1.41 (1.02–1.95)** *	1.11 (0.76–1.62)	413/88	1.31 (0.85–2.02)	1.31 (0.83–2.05)
Low	126/139	1.26 (0.86–1.84)	1.05 (0.67–1.65)	187/25	1.06 (0.59–1.89)	1.04 (0.56–1.91)

CM, cardiometabolic; SC, Sasang constitution; TE, Taeeum-in. Data are presented as the odds ratio (95% confidence interval). Model 1 was adjusted for sex and age. Model 2 was adjusted for sex, age, BMI, education, marriage, smoking, alcohol consumption, and disease history. * *p* < 0.05 and ^†^ *p* < 0.1. Bold font indicates statistical.

**Table 3:** The odds ratios of five cardiometabolic risk factors for each lifestyle based on SC type.

		High WC	High BP	High TG	Low HDL-C	High FPG
**TE group**	Physical activity (ref. = high)					
	Moderate	0.98 (0.64–1.5)	1.17 (0.8–1.7)	1.0 (0.69–1.44)	0.85 (0.59–1.24)	1.93 (1.06–3.51) *
	Low	1.7 (1.13–2.56) *	1.24 (0.87–1.78)	1.39 (0.99–1.96)	1.11 (0.78–1.58)	2.04 (1.14–3.63) *
	Sleep duration (ref. = 7 h)					
	≤6 h	1.12 (0.78–1.61)	0.86 (0.62–1.18)	0.99 (0.73–1.36)	0.75 (0.55–1.04)	1.51 (0.91–2.52)
	≥8 h	1.17 (0.73–1.9)	0.54 (0.34–0.86)	1.02 (0.67–1.57)	0.7 (0.45–1.09)	1.81 (0.92–3.53)
	Eating index (ref. = high)					
	Moderate	0.87 (0.58–1.3)	1.21 (0.83–1.77)	0.97 (0.67–1.39)	0.97 (0.68–1.39)	1.29 (0.73–2.27)
	Low	0.85 (0.52–1.38)	0.86 (0.55–1.35)	1.2 (0.79–1.83)	0.96 (0.62–1.49)	0.74 (0.36–1.51)
**Non-TE group**	Physical activity (ref. = high)					
	Moderate	1.1 (0.48–2.51)	0.92 (0.6–1.44)	1.4 (0.87–2.25)	1.76 (1.09–2.83) *	0.98 (0.4–2.41)
	Low	1.64 (0.75–3.58)	0.8 (0.52–1.23)	1.53 (0.97–2.41)	1.42 (0.88–2.29)	1.16 (0.51–2.66)
	Sleep duration (ref. = 7 h)					
	≤6 h	2.14 (1.01–4.5) *	1.04 (0.7–1.53)	1.29 (0.85–1.95)	1.45 (0.94–2.21)	1.13 (0.5–2.55)
	≥8 h	1.45 (0.56–3.74)	0.73 (0.42–1.25)	1.36 (0.79–2.33)	1.84 (1.09–3.1) *	1.9 (0.74–4.84)
	Eating index (ref. = high)					
	Moderate	1.47 (0.7–3.1)	1.36 (0.88–2.09)	1.85 (1.18–2.89) *	1.15 (0.76–1.74)	0.77 (0.35–1.72)
	Low	0.9 (0.32–2.48)	1.1 (0.62–1.92)	1.43 (0.8–2.57)	0.87 (0.49–1.54)	1.0 (0.35–2.82)

SC, Sasang constitution; TE, Taeeum-in; WC, waist circumference; BP, blood pressure; TG, triglyceride; HDL-C, high-density lipoprotein cholesterol; FPG, fasting plasma glucose. Data are presented as the odds ratio (95% confidence interval) adjusted for sex, age, BMI, education, marriage status, smoking habit, alcohol consumption, and disease history. * *p* < 0.05. Bold font indicates statistical.

## Data Availability

Not applicable.
